# Common Sense in the Use of Antiseptics

**Published:** 1897-10

**Authors:** Frank Blaisdell


### COMMON SENSE IN THE USE OF ANTISEPTICS.

The following incident came to the knowledge of the writer recently, and as it is a matter of common occurrence I will relate it:

A lady was about to have a laparotomy done at a prominent hospital; the surgeon was a young man and a good surgeon, and of course was well up in the use of antiseptics. The patient had been well prepared on the morning of the operation, and had been given the anæsthetic, and was thoroughly under its influence. It was time for the operation to begin, and yet the surgeon spent twenty minutes with a stiff brush, scouring the abdomen until it bled, and then took ten minutes more to sterilize his hands, thus consuming thirty minutes time which should have been given to the patient. This is more than wrong, for this thirty minutes belongs to the patient, and should have been given to her; and this thirty minutes in a critical operation might have turned the scale against the patient. I like the plan which I have carried out in the hospital under my charge, and it consists of having all the cleansing done on the morning of the operation, a sterilized pad applied, which the nurse removes when the surgeon takes up his knife. And the surgeon must be ready to operate the moment the patient is under the anæsthetic, for we believe that every moment saved to the patient is of direct influence upon the after effects of the operation. Antisepsis is the greatest boon yet given to the surgeon, and it behooves us to use it and not abuse it, and the best capital the surgeon or physician can have is an educated common sense.

FRANK BLAISDELL, M. D.

